# Rewards modulate saccade latency but not exogenous spatial attention

**DOI:** 10.3389/fpsyg.2015.01080

**Published:** 2015-07-28

**Authors:** Stephen Dunne, Amanda Ellison, Daniel T. Smith

**Affiliations:** Department of Psychology, Durham University, Stockton-on-Tees, UK

**Keywords:** saccade, instrumental, reward, learning, IOR, exogenous attention, oculomotor, premotor theory

## Abstract

The eye movement system is sensitive to reward. However, whilst the eye movement system is extremely flexible, the extent to which changes to oculomotor behavior induced by reward paradigms persist beyond the training period or transfer to other oculomotor tasks is unclear. To address these issues we examined the effects of presenting feedback that represented small monetary rewards to spatial locations on the latency of saccadic eye movements, the time-course of learning and extinction of the effects of rewarding saccades on exogenous spatial attention and oculomotor inhibition of return. Reward feedback produced a relative facilitation of saccadic latency in a stimulus driven saccade task which persisted for three blocks of extinction trials. However, this hemifield-specific effect failed to transfer to peripheral cueing tasks. We conclude that rewarding specific spatial locations is unlikely to induce long-term, systemic changes to the human oculomotor or attention systems.

## Introduction

The relationship between reward and behavior has become a central theme in psychology (Balleine and Dickinson, 1998), with an increasing number of studies focusing on the link between reward and eye movements. This association between reward and eye movements has been widely investigated, predominantly in primates using food rewards (e.g., Kawagoe et al., 1998; Takikawa et al., 2002; Bendiksby and Platt, 2006; Yamamoto et al., 2013). These studies show that saccades to rewarded locations are initiated earlier, have faster peak velocities and are more accurate relative to saccades to unrewarded locations. These behavioral changes are associated with profound changes in the subcortical structures that mediate saccadic eye movements, such that basal ganglia neurons are retuned to prefer the locations associated with reward (Kawagoe et al., 1998) and anticipatory activity in superior colliculus (SC) neurons that code the rewarded location is enhanced (Ikeda and Hikosaka, 2003, 2007). Studies have also revealed that presenting primates with two visual targets of varying reward values results in a gaze shift toward the higher value target (Coe et al., 2002; Milstein and Dorris, 2011) and that this bias in saccadic decision making is associated with modulation of target-related signals in lateral intraparietal area (LIP) (Platt and Glimcher, 1999). Recently Markowitz et al. (2011) investigated the time-course of integration of value information with the visual properties of a scene and reported that short-latency saccades (<∼150–180 ms) are driven by the properties of the scene (i.e., they are directed to the location with the highest salience), whereas longer latency saccades are biased toward the highest value locations, irrespective of salience.

Similar effects have been observed in human observers. For example, Milstein and Dorris (2007) used a monetary incentive such that participants were rewarded for fast and accurate prosaccades to a single visual target. The magnitude of reward was manipulated across left or right hemifields, such that one target location was associated with a higher reward than the target in the opposite hemifield. Consistent with the primate research, they observed a negative correlation between saccadic latency and reward, such that saccade latencies were faster to locations associated with larger rewards. Furthermore, oculomotor capture was greater when a distractor was presented at locations with a high expected value, suggesting the presence of saccade preparation toward high expected value locations prior to the onset of the movement goals. Theeuwes and Belopolsky (2012) also used an oculomotor task to examine whether a stimulus associated with high monetary reward has a greater ability to capture the eyes than the same stimulus when associated with a low reward. Participants were trained to associate one stimulus (a vertical line segment) with a high monetary reward and another stimulus (a horizontal line segment) with a low monetary reward. The amount of reward received was not related to participant performance, but instead was contingent upon the orientation of the target. During the test phase participant searched for a color singleton among an array of horizontal and vertical lines. Erroneous saccades to distractor items associated with large rewards were significantly more frequent than to low reward distracters. Furthermore, even when the stimulus no longer predicted reward, the learned value of the reward increased exogenous capture of the eyes above and beyond that driven by salience alone. Similarly, Bucker et al. (2015) observed that objects previously associated with a higher reward attracted the eyes in a stronger fashion than those associated with low or no monetary rewards. When rewards were no longer delivered, the bias found to higher-reward targets persisted. These data suggest that associating stimulus feature with a reward elicits a sustained bias in the oculomotor system. They also suggest that these rewards affect exogenous attentional capture by features. Consistent with this idea Stankevich and Geng (2015) found that pairing a stimulus feature with a reward produced an immediate attentional bias toward the rewarded color (as indexed by a manual reaction time). When the rewards were removed the magnitude of the bias was reduced but not entirely extinguished, suggesting that the rewards were able to produce a long-term effect on exogenous capture.

The finding that rewards modulate activity in the oculomotor system may have important implications for understanding how cognitive processes interact with the oculomotor system. For example, a number of authors have argued that the oculomotor system is critical for functions such as spatial attention (Rizzolatti et al., 1994; Schneider, 1995; Van der Stigchel and Theeuwes, 2005; Awh et al., 2006; Smith and Schenk, 2012), inhibition of return (IOR; Dorris et al., 2002; Fecteau and Munoz, 2005, 2006; Sereno et al., 2006), and spatial working memory (Awh et al., 1999; Pearson and Sahraie, 2003; Ikkai and Curtis, 2011; Ball et al., 2012; Pearson et al., 2014). If cognitive processes such as spatial attention are reliant on the oculomotor system, modulation of the oculomotor system with rewards should also modulate spatial attention. Furthermore, this finding offers the possibility that rewards may be used to help patients with brain injuries compensate for neuropsychological problems with attention and memory. Indeed, there is some recent evidence that rewarding object features can indeed attenuate some of the symptoms of deficits such as hemispatial neglect (Lucas et al., 2013; Malhotra et al., 2013). However, one reason to be cautious about applying spatial reward paradigms to neuropsychological patients is that very little is known about the time-course of the acquisition of learning, the time-course of extinction of learning or the extent to which learning transfers from the trained, oculomotor task to untrained cognitive tasks in neurotypcial participants.

To address these issues we examined the time-course of learning and extinction in a task where participants were rewarded for making a saccade to one of two potential target locations (Experiment 1). Based on the literature reviewed above, we predicted that saccades toward targets in the rewarded hemified would be significantly faster than those directed to the unrewarded hemifield during the learning phase. We then examined the effect of rewarding saccades on exogenous spatial attention and IOR, which refers to a bias against orienting to previously attended locations (Posner and Cohen, 1984). Here it was predicted that peripheral cues in the rewarded hemifield would produce greater attentional capture and IOR than those in the unrewarded hemifield. In this context it should be noted that the nature of IOR remains controversial. For example, some authors have argued that peripheral cues elicit two separable forms of inhibitory effect; a perceptual “inhibitory cueing effect” which delays target processing and a motor IOR effect which delays orienting to the cued location (Taylor and Klein, 1998; Sumner et al., 2004; Hilchey et al., 2014). In this view, only the oculomotor IOR effect is dependent on activity in the eye-movement system so this mode of IOR was the focus of study.

## Experiment 1

### Method

#### Participants

Twelve participants (10 female; 19–48 years; mean age 24.92 years) recruited from Durham University volunteered for the experiment and gave informed consent to participate. Ten were right eye-dominant. All participants had normal or corrected-to-normal vision and were naive regarding the purpose of the experiment. The study was approved by the Durham University Department of Psychology Ethics Committee.

#### Apparatus

The experimental stimuli were generated using a Cambridge Research Systems ViSaGe graphics card and displayed on a 17-inch Eizo Flexscan Color Display monitor with a refresh rate of 100 Hz. Responses were collected using a two-button button box. Eye movements were recorded using a Cambridge Research Systems eye tracker with a sampling rate of 250 Hz.

#### Stimuli

During the reward paradigm, participants were presented with a black (5 cm/2) 0.3° × 0.3° fixation cross in the center of the screen on a gray background (23 cm/2). A white target stimulus 0.5° × 0.5° (20 cm/2) square was presented to the left or right of the fixation cross. The stimuli were presented 6.5° to the left and 3.7° upward from fixation. After a rewarded trial participants were presented with reward feedback green text with a luminance of 19.61 cm/2 of “10p.” After an unrewarded trial participants were presented with reward feedback red text with a luminance of 19.69 cm/2 of “0p.”

#### Procedure

Participants were seated 57 cm away from the display with their head resting on a chinrest. A headband was placed around the top of the head to secure the participant’s head, controlling head movements. Participants underwent a 9-point calibration procedure prior to experimentation.

**FIGURE 1:**
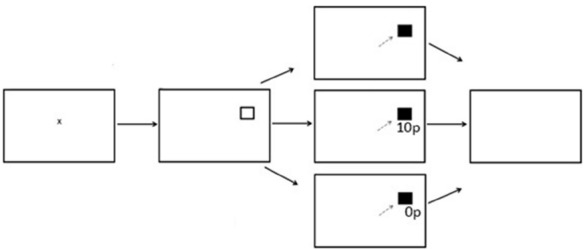
**Sequence of events used in Experiment 1 for the reward paradigm (not to scale).** The saccade goal was indicated by the appearance of a hollow square. When the target appeared at the rewarded location successful saccades received a reward of 10p on 60% of trials. There were no rewards when the target appeared at the unrewarded location.

There were three experimental phases; Preconditioning (two blocks, 120 trials), Conditioning (10 blocks, 600 trials) and Postconditioning (six blocks, 360 trials). Each block contained 60 trials with the entire reward paradigm lasting 18 blocks. Participants were instructed to fixate on the central fixation cross prior to the start of each trial. A fixation time period was programmed in which a lower limit of 500 ms and an upper limit of 800 ms was computed, followed by a target stimulus square in either the left or right hemifield. After a successful saccade the target stimulus would change color from black to gray. During the preconditioning phase of the experiment participants received no reward or reward feedback. During the conditioning phase of the experiment participants were rewarded for saccades made into only one hemifield. A variable-ratio reward schedule was employed. Of the 300 trials to the rewarded hemifield, 180 were rewarded (60%). On a rewarded trial, participants would receive additional information in the form of green text of “10p” presented in Arial font. On an unrewarded trial, red text of “0p” would be displayed below the original target stimuli. During the post-conditioning phase of the experiment, all reward was removed and participants would only receive feedback of red text of “0p,” regardless which hemifield the probe was presented to. Figure 1 displays the experimental array.

#### Statistical Analysis

The analysis was conducted on the mean of participant saccadic reaction time (SRT) averages calculated from each individual block. Data were filtered so that saccadic error and trials over 500 ms were eliminated from the analysis. The screen was divided into four quadrants and an erroneous saccade was classified as a saccade which landed in a quadrant that did not contain the saccade goal. In total, 1607 trials of all participants’ data (12.4%) were excluded from analysis.

### Results

#### Saccade Latency

**FIGURE 2:**
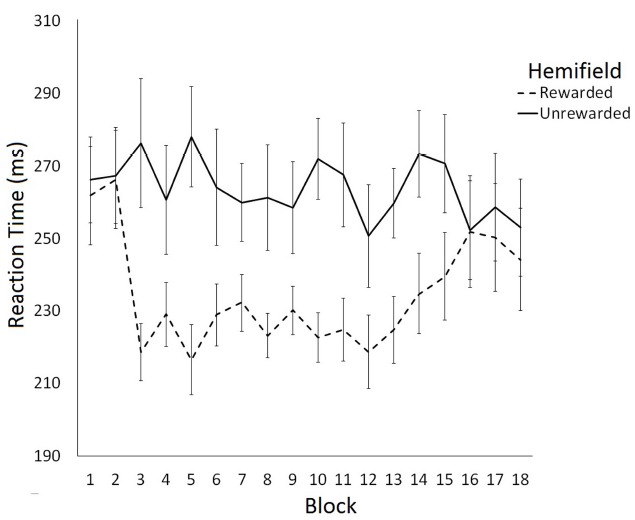
**Latency of prosaccades to the rewarded and unrewarded hemifields in Experiment 1 across the preconditioning, conditioning and post-conditioning phases.** Error bars show ±1 SEM.

The effect of rewarding a spatial location on saccade latency was examined using a 2 (Hemifield: Rewarded/Unrewarded) × 18 (Block: 1–18) repeated measures ANOVA on mean SRTs. This analysis revealed a main effect of Hemifield [*F*(1,11) = 7.78, *p* < 0.05] and a significant interaction between Block and Hemifield [*F*(2,17) = 1.85, *p* < 0.05]. Analysis of simple main effects revealed that saccade latencies toward targets in the unrewarded hemifield did not change as a function of block [*F*(1,17) = 0.77, *p* = 0.73]. In contrast, saccade latencies toward targets in the rewarded hemifield did change as a function on Block [*F*(1,17) = 2.53, *p* < 0.01]. A trend analysis of saccadic reaction times to the rewarded location indicated that the data were well fit by a quadratic function [*F*(1,17) = 6.69, *p* < 0.01], such that SRTs become faster after Block 2, then slowed down again after block 13. Trend analysis of saccadic reaction times to the unrewarded location revealed no linear or quadratic effects. Figure 2 illustrates these data. Consistent with our predictions facilitation of SRTs to the rewarded hemifield coincided with the introduction of the rewards in block 3. The facilitation dissipated between blocks 13 and 18, when rewards were no longer available.

To examine the time-course of extinction we conducted six further *post-hoc* two-tailed *t*-tests comparing SRTs to rewarded and unrewarded hemifields for blocks 13–18. This analysis revealed significant differences between the rewarded and unrewarded hemifield in the blocks 13 [*t*(11) = –2.71, *p* < 0.05, *r* = 0.40], 14 [*t*(11) = –2.81, *p* < 0.05, *r* = 0.65], and 15 [*t*(11) = –2.20, *p* = 0.05, *r* = 0.55] of the post-conditioning phase. However, these effects did not survive the Bonferroni correction for multiple comparisons.

#### Saccadic Error

Using the total proportion of errors a 2 (Hemifield: Rewarded/Unrewarded) × 18 (Block: 1–18) repeated measures ANOVA was conducted in order to assess whether the reward feedback had any effect on the proportion of participants errors. This analysis revealed no significant effect of Hemifield [*F*(1,11) = 0.16, *p* = 0.70], Block [*F*(17,187) = 2.20, *p* = 0.90] or interaction between Block and Hemifield [*F*(17,187) = 1.27, *p* = 0.21].

### Discussion

The aim of this experiment was to establish the extent to which changes in the metrics of saccades directed to a rewarded location persist once the rewards have been withdrawn. Consistent with previous studies, we observed significant facilitation for saccades directed toward the rewarded location (Milstein and Dorris, 2007) when rewards were available. However, our study also demonstrates a clear time-course for the acquisition and extinction of these effects. Firstly, the facilitation emerged after only one block of rewarded trials and the magnitude of the facilitatory effect was consistent across the conditioning phase. Secondly, during the post-conditioning phase facilitation of SRTs persisted for three blocks of trials, before returning to baseline levels. These data give a clear indication of the time-window in which we should expect to see modulation of exogenous covert orienting and IOR in Experiment 2.

## Experiment 2

### Method

#### Participants

Twenty-four new participants (16 female; 19–25 years; mean age 21 years) recruited from Durham University volunteered for the experiment and gave informed consent to participate. Seventeen were right eye-dominant. All participants had normal or corrected-to-normal vision and were naive regarding the purpose of the experiment. The study was approved by the Durham University Department of Psychology Ethics Committee.

#### Apparatus

The experimental stimuli were generated using a Cambridge Research Systems ViSaGe graphics card and displayed on a 17-inch Eizo Flexscan Color Display monitor with a refresh rate of 100 Hz. Responses were collected using a two-button button box. Eye movements were recorded using a Cambridge Research Systems eye tracker with a sampling rate of 250 Hz.

#### Stimuli

The reward paradigm was replicated from Experiment 2.

In the peripheral cueing task, participants were presented with a black 0.5° × 0.5° fixation cross in the center of the screen (0°) on a gray background with black 0.5° × 0.5° placeholders presented 8.0° to the left and right of the fixation cross. The cue was a white 0.5° × 0.5° square which appeared within one of the placeholders. The target was a 0.3° × 0.3° white target square.

#### Procedure

Participants were allocated to one of two groups. Group 1 (Exogenous attentional facilitation condition) received the target 150 ms after cue onset. Group 2 (IOR condition) saw the target 600 ms after cue onset. The same eye dominance test and calibration procedure outlined in Experiment 1 was replicated in the present experiment.

**Reward paradigm**

The reward paradigm was replicated from Experiment 1.

**Peripheral cueing task**

**FIGURE 3:**
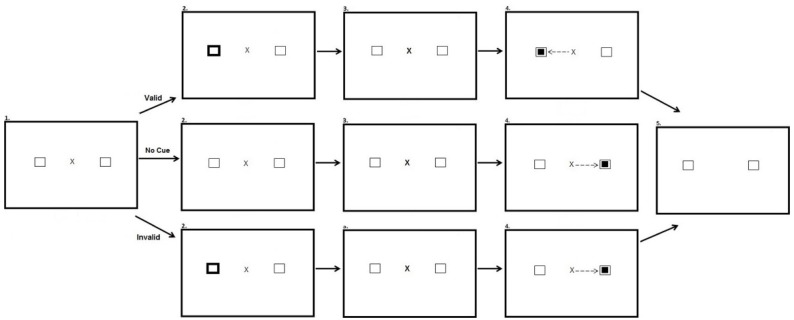
**Sequence of events used in Experiment 2 for the peripheral cueing task (not to scale).** Participants were presented with a fixation cross and two squares equidistant from the fixation cross in opposing hemifields (Row 1, Panel 1). In valid trials one of the squares changed color for a period of 100 ms, cueing participants to this location (Row 2, Panel 1). Participants are then presented with the same screen as in the first panel for a period of 50 ms (Row 3). A smaller target square then appeared in the same location as the cue, 100 or 600 ms after peripheral cue onset depending on the SOA manipulation, and participants were required to saccade to this location (Row 4, Panel 1). After making a successful saccade the fixation cross disappeared and the screen changed color requiring a button press to begin the next trial (Row 5, Panel 1). In no cue trials no cue appeared prior to target onset (Row 2, Panel 2). In invalid trials the cue appeared in one location (Row 2, Panel 3) and the target appeared in the opposite location (Row 4, Panel 3).

The peripheral cueing task occurred directly after the preconditioning, conditioning and post-conditioning phases of the reward paradigm. The experiment had three within participant factors: Experimental phase (After-preconditioning, After-conditioning, and After-postconditioning), Hemifield (Target in rewarded hemifield, Target in unrewarded hemifield), and Validity (Valid, Invalid and No Cue). There was also a between participants factor of stimulus onset asynchrony (SOA) (100 ms; 600 ms). Participants were presented with a fixation cross in the center of the screen and two black-outlined squares, one to the left and one to the right of fixation. On Valid and Invalid trials after 700 ms one of the black-outlined squares was cued by changing color from black to white for 100 ms. The fixation cross then pulsated for 50 ms to re-orient participant’s attention back to the center of the screen. Participants in the SOA:100 condition saw the target appear 150 ms after the onset of the peripheral cue. Participants in the SOA:600 saw the target appear 600 ms after the onset of the cue. On valid trials the target appeared at the same location as the cue. On invalid trials, the target appeared opposite the cued location. In no cue trials, the target was not preceded by a cue. Each block consisted of 60 trials equally split between each type of trial. The cue did not predict target location. Figure 3 illustrates the experimental array.

The experiment ran for 27 blocks and lasted for approximately 1 h. Participants switched between blocks of the two eye movement tasks. Firstly, participants completed the preconditioning phase of the reward paradigm (two blocks) and then the after-preconditioning of the exogenous orienting task (three blocks). Participants then completed the conditioning phase of the reward paradigm (10 blocks) followed by the after-conditioning phase of the exogenous orienting task (three blocks). Participants then completed the post-conditioning phase of the reward paradigm (six blocks) and finally the after-postconditioning phase of the exogenous orienting task (three blocks).

#### Saccade Analysis

The analysis was conducted on the means of each participant’s average SRT calculated from each individual block. Data was filtered so that saccadic error and trials over 500 ms were eliminated from the analysis; saccadic error refers to those trials in which saccades left the fixation area but did not land at the designated target location.

**Reward paradigm**

Across 25,290 trials, 3.6% were categorized as saccadic errors. 16.9% of trials were above the threshold and also removed from the analysis.

**Peripheral cueing task**

Of the 6,480 peripheral cueing task trials 4.95% were categorized as saccadic errors and 3.81% of trials were above the threshold and so removed from the analysis.

**Inhibition task**

Of the 6,480 inhibition task trials 13.12% were categorized as saccadic errors and 2.5% of trials were above the threshold and so removed from the analysis.

### Results

#### Latency

**Reward paradigm**

The effect of rewards on saccade latency were assessed with a 3 (Experimental Phase: Preconditioning/Conditioning/Post-Conditioning) × 2 (Hemifield: Rewarded/Unrewarded) × 2 (SOA 100/600) repeated measures ANOVA on mean saccadic reaction times. This analysis revealed a main effect of Experimental Phase [*F*(2,22) = 11.55, *p* = < 0.01, *r* = 0.59], such that saccades made during the conditioning phase (248 ms) where rewards were present were significantly faster than saccades made during the preconditioning (265 ms) [*t*(11) = 3.65, *p* = < 0.017, *r* = 0.74] and post-conditioning (268 ms) [*t*(11) = –5.72, *p* = < 0.017, *r* = 0.86] phases. There was no main effect of Hemifield [*F*(1,11) = 2.46, *p* = 0.15, *r* = 0.43], but there was a trend toward an interaction between Experimental Phase and Hemifield [*F*(2,22) = 3.04, *p* = 0.07, *r* = 0.35].

**FIGURE 4:**
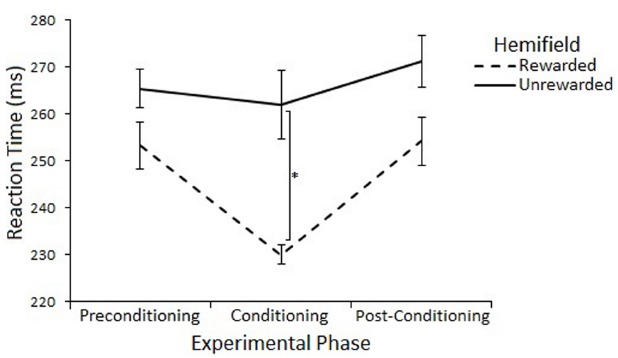
**Latency of prosaccades to the rewarded and unrewarded hemifields in Experiment 2 across the preconditioning, conditioning and post-conditioning phases.** Error bars show ±1 SEM. *p < 0.05.

Planned comparisons (two-tailed *t*-tests) were used to examine saccadic reaction time to rewarded and unrewarded locations at each level of Phase. There were no-significant differences in the preconditioning phase [*t*(11) = 0.01, *p* = > 0.017, *r* = < 0.01] or post-conditioning phase [*t*(11) = –0.69, *p* = > 0.017, *r* = 0.20] There was a significant differences between saccades to the rewarded (233 ms) and unrewarded (262 ms) hemifields during the conditioning phase [*t*(11) = –2.62, *p* = < 0.023, *r* = 0.62], but this effect does not survive a Bonferroni correction for multiple comparisons. Figure 4 illustrates this result.

**Peripheral cueing task**

In order to assess whether the effects of reward transferred to the peripheral cueing task a 3 (Experimental Phase: After Preconditioning/After Conditioning/After Post-Conditioning) × 2 (Hemifield: Rewarded/Unrewarded) × 3 (Validity: Valid/Invalid/No Cue) × 2 (SOA: 100/600) mixed model ANOVA on mean SRTs was conducted. This analysis revealed a main effect of Validity [*F*(2,22) = 15.91, *p* = < 0.05, ${\text{η}}_{p}^{2}$ = 0.42], a significant Validity × SOA interaction [*F*(2,22) = 25.25, *p* = < 0.05, ${\text{η}}_{p}^{2}$ = 0.53], a significant Validity × Phase interaction [*F*(2,22) = 2.79, *p* = < 0.05, ${\text{η}}_{p}^{2}$ = 0.11], and a significant Phase × Hemifield interaction [*F*(2,22) = 4.10, *p* = < 0.05, ${\text{η}}_{p}^{2}$ = 0.16].

The Validity × SOA interaction was explored by comparing Validity at each level of SOA with paired sample *t*-tests. At 100 ms SOA Valid trials were significantly faster than Invalid trials [308 vs 327, *t*(11) = 6.82, *p* < 0.017] but not No Cue trials [308 vs 301, *t*(11) = 2.10, *p* = 0.06]. Invalid trials were significantly slower than No Cue trials [326 vs 301, *t*(11) = 6.89, *p* < 0.05]. These data show attention facilitation for cued targets, although it is worth noting that this facilitation is driven primarily by slowing of SRTs to uncued targets, rather than facilitation of SRTs to cued targets. At 600 ms SOA Valid trials were significantly slower than Invalid trials [352 vs 332, *t*(11) = 4.18, *p* < 0.017] and No Cue trials [352 vs 331, *t*(11) = 6.06, *p* < 0.017]. Invalid trials were not significantly different than No Cue trials [331 vs 332, *t*(11) = 0.18, *p* > 0.05]. These data are consistent with IOR to cued targets.

The Validity × Phase interaction was explored by examining Phase at each level of Validity with ANOVA. There was no main effect of Phase on Valid trials [*F*(2,46) = 0.15, *p* = 0.86], a trend toward an effect on No Cue trials [*F*(2,46) = 2.77, *p* = 0.073] and a significant main effect on Invalid trials [*F*(2,46) = 5.68, *p* < 0.05]. *Post-hoc* tests (paired samples *t*-tests) show that SRTs invalidly cued were significantly faster in the conditioning phase compared to the preconditioning phase [322 vs 336, *t*(23) = 2.92, *p* < 0.017] and the post-conditioning phase [322 vs 331, *t*(23) = 2.76, *p* < 0.017]. There was no difference between the preconditioning and the post-conditioning phases [336 vs 331, *t*(23) = 1.06, *p* > 0.017].

**FIGURE 5:**
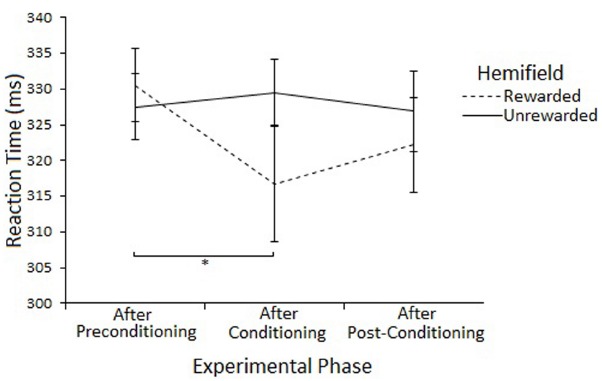
**Latency of prosaccades to the rewarded and unrewarded hemifields in the peripheral cueing of Experiment 2 across experimental phases.** Error bars show ±1 SEM. *p < 0.05.

The Hemifield × Phase interaction was explored by examining Phase at each level of Hemifield with ANOVA. There was a main effect of Phase on Rewarded trials [*F*(2,46) = 3.74, *p* < 0.05] but not on Unrewarded trials [*F*(2,46) = 0.31, *p* = 0.73]. *Post-hoc* tests (paired samples *t*-tests) suggest that SRTs to targets in the Rewarded hemifield were faster in the conditioning phase compared to the preconditioning phase [316 vs 330, *t*(23) = 2.27, *p* = 0.033] but not the post-conditioning phase [316 vs 322, *t*(23) = 1.15, *p* = 0.261]. There was no difference between the preconditioning and the post-conditioning phases [330 vs 322, *t*(23) = 1.99, *p* = 0.058]. However, none of the effects survive Bonferroni correction. Figure 5 illustrates this result.

We conducted a final analysis to examine whether participants made a greater number of anticipatory errors toward the rewarded hemifield. A 3 (Experimental Phase: After Preconditioning/After Conditioning/After Post-Conditioning) × 2 (Hemifield: Rewarded/Unrewarded) repeated measures ANOVA with a between-subjects factor of SOA was conducted on the number of anticipatory eye-movements made during the peripheral cueing task. This analysis revealed no significant effects or interactions suggestive that rewards failed to have an effect on participant’s anticipatory behavior.

### Discussion

Experiment 2 confirmed that rewards facilitate saccade latencies during the conditioning phase. There was also evidence that saccades to the rewarded hemifield were facilitated during the peripheral cueing task, demonstrating that the training task successfully modulated the oculomotor system and that this modulation persisted during the peripheral cueing task. However, there was no evidence that this effect of reward interacted with covert attention. Specifically, we observed no three-way interaction between Hemifield, Validity, and Phase. Similarly, although a significantly larger number of anticipations were made toward the rewarded location, the lack of an interaction between Phase and Validity suggests that these anticipations were not modulated by reward. The SOA × Validity interaction is consistent with the biphasic effects of peripheral cues, which produce attentional facilitation at short SOAs and IOR at long SOAs (Posner and Cohen, 1984). The Validity × Phase interaction suggests that the introduction of the reward had a non-spatially specific effect of facilitating response times on invalid trials. This may reflect a generalized alerting effect of the reward, which allowed participants to react more quickly to the appearance of the target at the uncued location (i.e., the rewards helped reduce the cost of attending to the wrong location).

Overall the data in this experiment confirm that rewarding spatial locations produces significant modulation of the oculomotor system, as evidenced by the facilitation of saccadic reaction time. This modulation can also be observed in a related task which also requires eye-movements but does not have a reward component. Importantly, however, we have also demonstrated that the modulation of the oculomotor system does not interact with covert spatial attention. From a theoretical perspective this is an important point, as it suggests that it is possible to modulate the oculomotor system without affecting covert attention, contrary to the predictions of the premotor theory of attention (Rizzolatti et al., 1994; Smith and Schenk, 2012)

## General Discussion

Experiments 1 and 2 show a significant facilitation of saccadic reaction time for eye-movements directed to a rewarded location. These data are consistent with evidence of the effect of reward on the oculomotor system in both primates and humans (Bowman et al., 1996; Coe et al., 2002; Takikawa et al., 2002; Bendiksby and Platt, 2006; Milstein and Dorris, 2007, 2011). In an important extension of previous work, Experiment 1 examined the time-course of extinction of this facilitation. This study suggested that the facilitation persisted for a short period of time (three blocks). Experiment 2 examined to what extent the facilitation transferred to untrained tasks that are also hypothesized to engage the oculomotor system (exogenous attention and IOR). The facilitation of saccades toward the rewarded hemifield was sustained, but this effect did not interact with either exogenous attentional facilitation or IOR.

The finding that rewards modulated saccade latency in a stimulus-driven saccade task but not exogenous orienting or IOR can be accounted for in terms of accumulator models of saccade production (e.g., LATER—Carpenter and Williams, 1995; Findlay and Walker, 1999; Munoz and Schall, 2003). In these models saccade generation is determined by two factors. The first factor is the relative distance between baseline activation and execution thresholds. The second is the rate at which evidence that a particular location is the saccade target is accumulated. Research in primates suggests that the oculomotor neurons that represent the location of an expected reward exhibit a heightened activity level (Platt and Glimcher, 1999; Dorris and Glimcher, 2004). In principle this elevated neuronal activity is equivalent to a shift in the baseline activity level. This heightened baseline activation means the distance between baseline and execution threshold is reduced, resulting in faster saccadic reaction times to the rewarded location. The facilitation of SRTs to rewarded locations was also observed in the peripheral cueing task, suggesting that this baseline-shift was also present during the cueing task. However, this baseline shift did not interact with exogenous attention or IOR.

The finding that modulation of the eye-movement system did not interact with covert attention was somewhat surprising, particularly given the strong evidence that exogenous attention (e.g., Smith et al., 2010, 2012b, 2014; Morgan et al., 2014) and IOR (e.g., Hilchey et al., 2014) depends on the eye-movement system. However, this result is consistent with other studies which argue for dissociation between the oculomotor system and covert, exogenous spatial orienting (Hunt and Kingstone, 2003; MacLean et al., 2015). One way to explain this apparent dissociation is to propose that that rewards and covert attention interact with different components of the oculomotor system. Specifically, rewards appear to act on execution threshold (see above) whereas attention may operate on the accumulation rate (Smith, 2000; Carrasco and McElree, 2001). If this proposal is correct, rewarding one hemifield would facilitate reaction times (RTs) for all targets appearing in that hemifield, irrespective of their validity. One way to test this theory is to examine whether the effects of rewarding eye-movements to a specific location generalize to manual reaction times on cueing tasks. Here, it would be predicted that the rewards would not elicit hemisphere-specific facilitation of manual reaction times because the rewards are operating on the thresholds for the initiation of a saccadic eye-movement, not the threshold for the initiation of a button press.

The facilitation of SRTs observed in the reward paradigm also failed to transfer to the IOR task In studies of perceptual IOR the impaired perception at cued locations occurs because the sensory processing of visual signals arising from the cued location is suppressed (Muller and Kleinschmidt, 2007; Dukewich, 2009; Prime and Jolicoeur, 2009; Smith and Schenk, 2010; Smith et al., 2012a; Sapir et al., 2014). The mechanism underpinning saccadic IOR is less clear. One might suppose that saccadic IOR occurs because the oculomotor activity related to the cued location is suppressed. However, this line of argument is not consistent with the neurophysiological evidence, which suggests that peripheral cues modulate the sensory, not motor responses in oculomotor structures such as the superior colliculus (Dorris et al., 2002; Fecteau and Munoz, 2005). Ludwig et al. (2009) have argued that saccadic IOR occurs as the consequence of a reduction in the accumulation rate of activity related to the target location (rather than a change in the execution threshold for saccades to the target location). Suppressing the visual signals arising from a cued location would have the effect of reducing the accumulation rate. This line of argument suggests that perceptual and saccadic IOR are instantiated by a common neurophysiological mechanism, specifically, a reduction in the quality of sensory information relating to new sensory events at a cued location and not the suppression of motor activity related to the cued location. This reduced quality of sensory information produces slower detection and discrimination in manual RT tasks, and increases the time needed to produce a stimulus driven eye-movement in saccadic RT tasks. Our finding that rewards modulate activity in the oculomotor system without affecting IOR is consistent with the view that IOR primarily acts on the sensory, not motor system.

In summary, it has been found that rewarding spatial locations can facilitate the latencies of eye movements reproduced across three experiments, confirming previous work in both primates and humans. This study also extended previous findings by establishing a time-course for the effects of reward in human observers. Experiment 2 utilized this time-course to examine the extent to which learning in the oculomotor system transferred to cognitive tasks known to engage the oculomotor system, specifically exogenous attentional orienting and IOR. These studies revealed that the facilitation of saccadic reaction times toward rewarded locations did persist in the transfer task. However, this modulation of the oculomotor system did not interact with exogenous orienting of spatial attention or IOR. We conclude that (a) exogenous attentional facilitation can be decoupled from the oculomotor system, contrary to the premotor theory of attention (Smith and Schenk, 2012) (b) IOR arises from changes in the sensory processing of the signals arising from the cued location, rather than changes in the motor activity relating to the cued location and (c) that rewarding eye movements to specific spatial locations is unlikely to induce long-term, systemic changes to the human eye movement or attention systems. Given this limitation it may not be a viable tool in the alleviation of symptoms associated with neuropsychological visual deficits.

### Conflict of Interest Statement

The authors declare that the research was conducted in the absence of any commercial or financial relationships that could be construed as a potential conflict of interest.
